# Neutralizing Antibodies vs. Viruses: Interacting Mechanisms and Escape Tactics

**DOI:** 10.3390/microorganisms13092199

**Published:** 2025-09-19

**Authors:** Hao Lu, Yichen Liu, Yue Song, Longxin Chen, Limeng Zhang, Runting Li, Xiaoning Nie, Guoqiang Zhu, Xueyan Ding, Linqing Wang

**Affiliations:** 1 Molecular Biology Laboratory, Zhengzhou Normal University, Zhengzhou 450044, China; 2 College of Veterinary Medicine, Henan Agricultural University, Zhengzhou 450046, China; 3 College of Veterinary Medicine, Yangzhou University, Yangzhou 225009, China; 4 Department of Life Science, Zhengzhou Normal University, Zhengzhou 450044, China

**Keywords:** VNAs, antiviral immunity, immune escape, broad-spectrum antibody, challenges

## Abstract

Virus-neutralizing antibodies (VNAs) serve as critical components of host immune defense, countering viral infections by specifically recognizing epitopes on viral surface antigens to block viral entry and replication. This review elucidates the functional mechanisms of VNAs, with a focus on the dynamic interactions between the Fab region and viral epitopes, including steric hindrance and conformational locking, as well as the effector functions mediated by the Fc segment. Furthermore, we dissect diverse viral evasion strategies against neutralization that have emerged in recent studies, encompassing antigenic drift/shift, glycan shielding, epitope occlusion, antibody-dependent enhancement, and mutation accumulation under population immune pressure. Integrating structural biology insights with clinical evidence, we analyze challenges in developing broadly neutralizing antibodies and highlight innovative technological approaches. Our synthesis aims to establish a theoretical framework for the rational design and clinical translation of next-generation VNAs, thereby advancing novel strategies for antiviral therapeutics development.

## 1. Introduction

Throughout biological evolution, the dynamic interplay between viruses and their hosts constitutes an incessant molecular arms race. Virus-neutralizing antibodies (VNAs) serve as the primary defense armament of the immune system, leveraging their exquisite molecular specificity to fulfill irreplaceable roles in both innate and adaptive immunity [1]. Defined by their capacity to bind directly to viral surface proteins—such as spike, envelope, or capsid proteins—VNAs disrupt viral attachment, entry, or replication [2,3]. Through high-fidelity recognition of surface antigenic epitopes, they directly block host cell invasion or suppress viral propagation. Antibodies represented by IgG, IgA, and IgM serve as the primary effector molecules of adaptive humoral immunity, capable of precisely recognizing and counteracting pathogens.: the Fab region mediates antigen engagement, while the Fc domain orchestrates immune effector functions. Particularly against both enveloped and non-enveloped viruses, VNAs demonstrate potent antiviral efficacy via mechanisms such as steric hindrance and conformational immobilization, establishing their status as the cornerstone of monoclonal antibody therapeutics and vaccine development [3].

Currently, the preparation strategies for VNAs are becoming increasingly diversified. One approach involves isolating specific memory B cells from the peripheral blood of convalescent patients and directly cloning the naturally paired fully human heavy and light chain genes using single-cell PCR technology [4]. Alternatively, in vitro display technologies such as phage display, yeast display, or ribosome display can be employed to conduct high-throughput screening in artificial antibody libraries with scales ranging from 10^9^ to 10^11^, thereby identifying high-affinity clones [5]. Additionally, human antibody transgenic mouse models can be utilized, where immunization induces in vivo affinity maturation to generate monoclonal antibody candidates with sequences highly similar to those of natural human antibodies [6,7]. The clinical potential of VNAs has gained significant prominence in combating infectious diseases in recent years. Breakthrough applications in emergency therapeutics and post-exposure prophylaxis are exemplified by the REGN-COV2 antibody cocktail during the COVID-19 pandemic and the Ebola virus monoclonal antibody mAb114 [8,9]. Nevertheless, evolving viral escape mechanisms—including high-frequency mutations (antigenic drift/shift), glycan shielding, and conformational dynamics of epitopes—continuously compromise neutralizing efficacy, presenting a fundamental research challenge. Notably, spike protein mutations in the Omicron variant and extensive glycosylation of the HIV Env protein have substantially hindered the development of broadly neutralizing antibodies [10,11]. Against this backdrop, overcoming these evolutionary barriers to achieve long-lasting potency and broad-spectrum coverage in antibody therapeutics has emerged as a pivotal challenge at the immunology–structural biology interface.

We synthesize the molecular mechanisms of the action of VNAs and comprehensively dissect multifaceted viral escape strategies—including epitope occultation, antibody-dependent enhancement (ADE), and mutation accumulation under population immune pressure—while discussing cutting-edge optimization approaches such as AI-driven antibody engineering, multi-specific antibody design, and novel delivery platforms. Crucially, we integrate conformational dynamics revealed by cryo-electron microscopy (cryo-EM), AI-predicted conserved epitopes, and clinical translation case studies. By unifying the structural–functional principles of VNAs, viral evasion mechanisms, and associated challenges with emerging innovations, this work establishes a dual-pronged framework encompassing theoretical foundations and actionable development roadmaps. Our synthesis aims to catalyze synergistic advancements in precision medicine and interdisciplinary immunology–engineering convergence for next-generation VNAs development.

## 2. The Action Mechanisms of VNAs

### 2.1. Direct Neutralization Mechanisms

VNAs constitute a core defensive strategy bridging innate and adaptive immunity by disrupting critical viral entry steps through targeted engagement of functional surface proteins. These mechanisms depend not only on antibody structural architectures but also on epitope accessibility, conformation dynamics, and host–pathogen interfacial interactions. Notably, virus lysis or aggregation induced by antibodies such as 8C11 and ZIKV-117 constitutes an irreversible process; even after antibody dissociation, the virus permanently loses its infectivity [12,13]. Here, we synthesize recent advances to delineate the multifaceted modes and molecular foundations underpinning direct VNA neutralization mechanisms.

#### 2.1.1. Mechanisms Targeting Viral Entry

##### Steric Blockade of Receptor-Binding Sites

**Figure 1:**
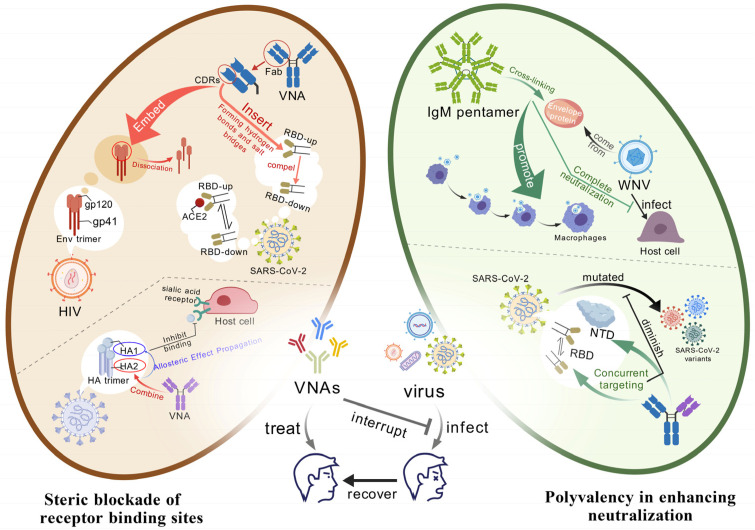
**The mechanism of VNAs against viral entry.** VNAs mainly target the invasion of viruses through the following two pathways: (1) Spatial blocking-high-affinity Fab segments are embedded into key viral epitopes in a “key-lock” manner, forming a physical barrier and blocking receptor-binding sites; the triple molecular actions led by CDR loops include hydrogen bond network reconstruction (the CDR H3 of REGN10987 pushes the SARS-CoV-2 spike RBD from “up” to “down”), hydrophobic core perturbation (the CDR H2 of VRC01 disrupts the HIV gp120-gp41 interface through π-π stacking and hydrophobic insertion), and allosteric signal transduction (CR8020 binding to the HA stem remotely inhibits sialic acid binding). (2) Polyvalent synergy—the IgM pentamer simultaneously cross-links the West Nile virus E protein with 10 Fab segments, not only enhancing the rigidity of the capsid to prevent receptor approach but also accelerating virus opsonophagocytosis; the bispecific antibody SA55 simultaneously locks the SARS-CoV-2 spike RBD and NTD, achieving dual spatial blockade and delaying escape mutations.

Steric blockade represents the canonical mechanism of antibody neutralization, wherein high-affinity Fab binding to critical functional domains of viral surface proteins physically occludes host receptor engagement. At the molecular level, complementarity-determining regions (CDRs) exploit multivalent interactions to induce epitope conformational changes, manifested through hydrogen bond network remodeling, hydrophobic core perturbation, and allosteric signal propagation (Figure 1).

CDRs establish specific binding interfaces via hydrogen bonding networks. SARS-CoV-2-neutralizing antibody REGN10987, for instance, inserts its CDR H3 loop into the ACE2-binding site of the receptor-binding domain (RBD). Tyr102 forms a pivotal hydrogen bond with Gly485, while Asp99 engages Asn487 via salt bridge formation [14,15]. These interactions trigger a 2.3 Å displacement in β-sheet topology, forcing RBD transition from an “up” (open) to “down” (closed) conformation that disrupts ACE2 binding [16,17]. Concurrently, CDRs destabilize viral proteins through hydrophobic interactions. The HIV broadly neutralizing antibody VRC01 penetrates the CD4-binding pocket of Env gp120 via its CDR H2 loop: Phe54 establishes π-π stacking with Trp427, while Leu100 inserts into a hydrophobic cavity formed by Asp368 and Gly367. This disrupts native gp120-gp41 interactions, ultimately triggering trimer dissociation [18,19].

Allosteric propagation enables antibody binding to induce cooperative conformational changes in distal domains. Influenza HA-stem antibody CR8020 binds near the fusion peptide in HA2, transmitting allosteric signals to HA1′s RBD via β-sheet hydrogen bond rearrangement, thereby inhibiting sialic acid receptor engagement [20,21]. Similarly, antibody FI6v3 maintains neutralizing potency against H3N2 antigenic drift (e.g., E62K mutation) by targeting conserved fusion-peptide-proximal residues, demonstrating allostery’s robustness against viral evolution [22,23]. Collectively, these mechanisms reveal that antibodies neutralize pathogens not merely through steric hindrance but via molecular-force-driven conformational remodeling of viral proteins—achieving both potent and broad-spectrum efficacy.

##### Polyvalency in Enhancing Neutralization

Beyond conventional steric blockade, certain VNAs leverage multivalent binding sites to enhance affinity and neutralization potency through cooperative antigen engagement—a mechanism of particular significance against rapidly evolving viruses. This enhancement operates through two distinct yet interconnected dimensions (Figure 1):

Spatially, IgM pentamers form cross-linking networks via simultaneous engagement of repetitive viral epitopes by ten Fab domains [24]. Anti-West Nile virus IgM (E16) accelerates viral phagocytosis by mononuclear macrophages through E-protein cross-linking [25]. Small-angle X-ray scattering (SAXS) analyses reveal that such multivalent binding compresses E-protein dimer spacing from 4.2 nm to 3.1 nm, rigidifying the viral surface and completely occluding receptor access [26]. Single-molecule studies further demonstrate that Zika-neutralizing antibody ZIKV-117 induces irreversible aggregation by bridging E-dimers across viral particles, abolishing infectivity [25].

Conformationally, bispecific antibodies trigger allosteric communication through cooperative multi-epitope engagement. SARS-CoV-2 antibody SA55, for instance, concurrently targets the RBD and N-terminal domain (NTD) of the spike trimer. NTD binding (residues 14–305) induces β-barrel rearrangement, while RBD engagement collectively elevates the spike’s conformational free-energy barrier by 1.8-fold [27]. This dual-lock mechanism impedes viral escape mutants—a paradigm shift from traditional mono-epitope targeting that informs broad-spectrum antiviral design.

##### Inhibition of Conformational Changes Required for Fusion

**Figure 2:**
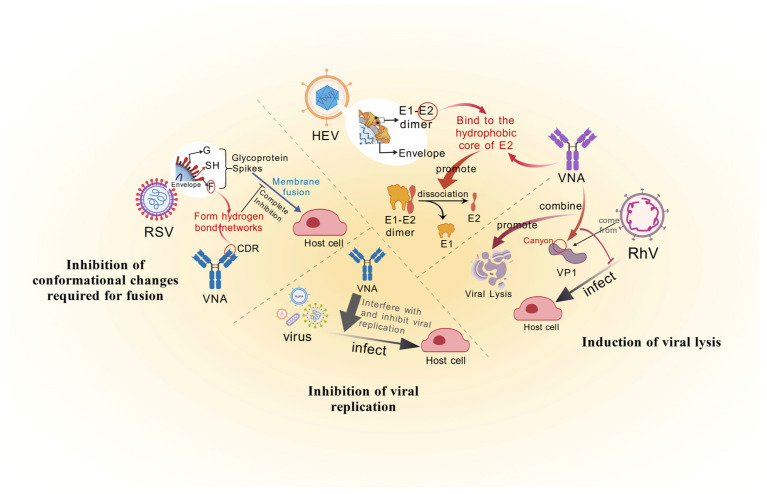
**The conformational changes caused by VNAs inhibit and lead to virus lysis.** Conformational locking and viral lysis—some VNAs induce viral proteins to “freeze” in an inactive state, such as nirsevimab precisely clamping the RSV F protein, blocking its transformation from the prefusion to the postfusion conformation; other antibodies directly trigger viral disintegration: Hepatitis E Virus (HEV) 8C11 inserts into the hydrophobic core of E2, pulling apart the dimer interface; Rhinovirus (RhV) 8F12 binds to the “canyon region” of VP1, blocking receptor recognition and causing premature capsid disintegration; and some antibodies can target non-structural proteins and interfere with host–virus interactions.

Notably, selected VNAs achieve irreversible functional disruption by inducing conformational locking of viral proteins—a mechanism not restricted to specific viral contexts but conserved across diverse pathogens. This paradigm reveals fundamental principles of antibody-mediated neutralization and informs next-generation antiviral strategies (Figure 2).

*Freezing of the pre* *-fused state*

In respiratory syncytial virus (RSV), the antibody nirsevimab precisely engages specific antigenic sites (residues 62–69 and 196–209) of the fusion (F) glycoprotein, arresting its prefusion-to-postfusion transition [28]. Cryo-electron tomography (cryo-ET) reveals a critical mechanistic detail: stabilization of the prefusion conformation occurs through hydrogen bond networking between nirsevimab’s CDR L1 loop and the α4 helix of F. This interaction elevates the activation energy barrier by 4.2 kcal/mol [29], thereby fully suppressing the conformational rearrangements required for membrane fusion.

*The “obstruction” formed by the fusion holes*

In Ebola virus, antibody ADI-15878 binds a densely packed hydrophobic pocket within the GP base (residues 40–150), disrupting hydrogen bonds and hydrophobic interactions between GP1 and GP2 subunits [30]. This impairment prevents assembly of the six-helix bundle (6-HB)—a structure essential for fusion pore formation [31]—thereby abolishing viral membrane fusion capability.

In summary, the steric blocking mechanism of VNAs is fundamentally driven by antigen–antibody interaction-induced conformational rearrangements of viral surface proteins [32]. Such binding may trigger functional inactivation of viral proteins, resulting in loss of infectivity. Beyond physically occluding viral entry into cells, certain antibodies can promote premature conformational transitions in metastable viral proteins—exemplified by stabilizing the SARS-CoV-2 spike protein in a postfusion state—thereby expediting viral inactivation [33,34]. Compared to mere steric shielding, such conformation-modulating mechanisms achieve neutralization more efficiently and provide critical structural biological insights for the rational design of antibody therapeutics resistant to viral variants [35].

#### 2.1.2. Lysis and Replication Inhibition of Viruses

VNAs confer protection not only by blocking viral entry but also through direct virion disruption or replication inhibition—mechanisms particularly critical for non-enveloped viruses and select enveloped pathogens (Figure 2).

##### Induction of Viral Lysis

Certain VNAs trigger conformational rearrangements or mechanical stress upon binding key epitopes on viral capsids/envelope proteins, ultimately inducing virion lysis [36]. For instance, the HEV-neutralizing antibody 8C11 binds the hydrophobic core of the E2 capsid protein, provoking dimer interface dissociation that culminates in viral disintegration [12]. Similarly, anti-RhV Fab 8F12 engages the VP1 canyon region, simultaneously blocking host–receptor interactions [37] and inducing capsid rigidification that mechanically precipitates premature disassembly during endosomal trafficking [38].

##### Inhibition of Viral Replication

Other VNAs target non-structural viral proteins (e.g., polymerases or proteases) or indirectly impede replication by disrupting host–pathogen interplay [39]. Exemplifying this, DENV-targeting mAb 2H5 binds the NS1 membrane-binding domain, preventing its association with host lipid rafts and thereby suppressing virion assembly/release [40]. Concurrently, the HIV integrase-neutralizing antibody VRC07-523LS occupies the enzyme’s catalytic core domain, obstructing viral DNA integration into the host genome [41]. Critically, such antibody engineering must avoid host homolog cross-reactivity to minimize off-target risks.

### 2.2. Indirect Effect Functions

VNAs amplify antiviral immunity through indirect effector functions—mediated by Fc domain engagement with host immune components—which activate diverse clearance mechanisms to broaden immune coverage and enhance durability. This process is governed not only by antibody structural features (glycosylation profiles, subclass distributions, and valency states) but also by dynamic virus–host interplay [42]. Below, we dissect the molecular mechanisms underpinning Fc-driven immune clearance and its synergy with mucosal defenses.

#### 2.2.1. Fc-Mediated Immune Clearance

**Figure 3:**
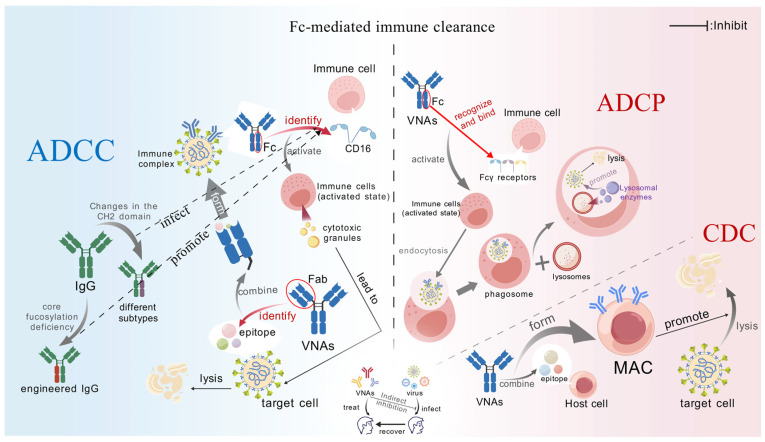
**Fc-mediated immune clearance.** The Fc-mediated immune clearance mechanism of VNAs mainly includes the following three types. (1) After VNAs-Fab bind to the target antigen, the Fc portion interacts with the FcγRIIIa of NK cells, triggering the release of granzyme/perforin; the affinity of IgG1 is greater than that of IgG3, and the removal of fucose further enhances the effect. (2) The Fc of ADCP binds to the FcγR (RI/IIa/IIIa) of macrophages, inducing the formation of phagosomes and their fusion with lysosomes; Fc engineering (point mutation/glycosylation) enhances the phagocytic efficiency. (3) The Fc-C1q binding initiates the complement cascade, generating the MAC to lyse the virus or infected cells.

The Fc segment of antibodies, upon binding to immune cell surface receptors (e.g., FcγR, FcαR) or complement components, can activate immune effector mechanisms such as ADCC, ADCP, and CDC (Figure 3).

Recent in vivo studies have further quantified the contribution of the Fc effector function in controlling viral infections. Existing evidence indicates that even if the Fab segment of the antibody completely loses its neutralizing activity and fails to block viral entry, the antibody can still effectively clear infected cells, reduce viral load, and modulate the inflammatory microenvironment through pathways such as ADCC and ADCP, provided its Fc segment exhibits high affinity for activating FcγR (particularly FcγRIIIa) [43,44,45]. For instance, in dengue fever animal models, certain antibodies, despite being unable to block viral entry, significantly reduced viral load due to robust Fc effector functions [46].

It is noteworthy that excessively strong Fc functionality or low antibody concentrations may trigger ADE, facilitating viral evasion [47]. Therefore, an ideal therapeutic antibody should achieve a balance between Fab-mediated neutralization and Fc-mediated immune clearance rather than relying solely on a single mechanism.

##### ADCC

ADCC eliminates pathogens or infected cells through antibody-mediated cytotoxic immune responses. VNAs initially bind target-cell antigens via their Fab domains. Subsequent Fc engagement of Fcγ receptors (e.g., FcγRIIIa/CD16) on effector cells—notably natural killer (NK) cells—triggers cytotoxic granule release (perforin/granzymes), directly lysing bound targets [48]. Key regulatory determinants include the following:

*IgG Subclass Specificity*

IgG subtypes exhibit markedly distinct FcγRIIIa affinities, directly modulating ADCC efficiency. IgG1 demonstrates higher affinity (KD ≈ 100 nM) than IgG3 (KD ≈ 300 nM) due to CH2 domain conformational stability—a divergence rooted in amino acid composition and structural dynamics [49,50]. For SARS-CoV-2 antibodies, IgG1 and IgG3 differentially mediate antibody-dependent cell-mediated virus inhibition through combinatorial effects of antibody specificity and subclass properties [51].

*Glycosylation modification*

Afucosylation substantially enhances FcγRIIIa binding and ADCC potency [52]. Ebola antibody REGN-EB3 engineered for reduced core fucose elevated NK-mediated killing by 70%, correlating with 92% clinical efficacy [53]. Leila et al. demonstrated that glycoengineered variants of HIV bNAb 10-1074—exhibiting distinct glycoform profiles (varying fucose/galactose content)—achieved differential ADCC activity, with afucosylated forms showing maximal enhancement [54].

##### ADCP

ADCP represents a pivotal defense mechanism wherein FcγR-bearing immune cells phagocytose antibody-opsonized targets. VNAs bridge pathogens to phagocytes (e.g., macrophages) via FcγR interactions (FcγRIIa/RI/IIIa), inducing phagocytic activation. Immune cells internalize bound targets into phagosomes [55], which fuse with lysosomes to release degradative enzymes (e.g., perforin/granzymes), eliminating internalized pathogens.

In addition to enhancing ADCC efficiency as previously mentioned, engineered modifications to the Fc region can significantly augment ADCP [56]. Through site-directed amino acid mutations and glycosylation modifications, the affinity of antibodies for activating Fcγ receptors can be specifically enhanced, thereby boosting their phagocytic activity [57]. Furthermore, Fc engineering strategies also offer multiple advantages, including reducing the risk of ADE and prolonging serum half-life [58,59]. With the advancement of protein engineering technologies, rational design of the Fc domain will provide critical insights for the multifunctional development of next-generation antibody therapeutics.

Furthermore, the pentameric structure of multimeric IgM plays a critical role in enhancing ADCP efficiency through synergistic mechanisms. This multivalent architecture enables IgM to bind multiple antigenic epitopes simultaneously via its Fab regions, promoting the formation of high-density immune complexes [60]. One mechanism by which IgM enhances ADCP involves the engagement of its Fc region with the Fcμ receptor (FcμR) expressed on macrophages and dendritic cells (DCs) [61]. This FcμR binding potentiates immune cell recognition and engulfment of opsonized targets. Alternatively, IgM’s multivalency facilitates polyvalent cross-linking of antigens, which significantly augments the phagocytic efficiency of effector cells [62]. This functional enhancement is exemplified by studies demonstrating that neutralizing antibodies engineered into the IgM format exhibit substantially increased viral neutralization potency compared to their IgG counterparts [63].

##### CDC

CDC eliminates target cells (e.g., cancer cells or pathogen-infected cells) through antibody-mediated activation of the classical complement pathway, culminating in membrane attack complex (MAC) formation. The complement system—comprising ≈30 soluble and membrane-bound proteins—constitutes a critical immune component that identifies and eliminates pathogens, apoptotic cells, and cellular debris [64]. When VNAs bind surface antigens, their Fc domains engage complement component C1q, triggering the classical pathway. This cascade assembles MAC (C5b-9 complexes) that inserts into cell membranes or viral envelopes, inducing osmotic lysis via pore formation and content leakage [65].

Notably, antibody subtypes exhibit marked efficiency differences: IgG3 demonstrates 5-fold greater CDC activity than IgG1 due to extended hinge regions exposing additional C1q-binding sites [66]. For instance, reformatting anti-HSV-1 antibody HDIT101 to IgG3 elevated complement-mediated viral lysis from 40% to 85%, directly attributable to enhanced C1q avidity [66,67].

Furthermore, inflammatory conditions augment CDC through host protease activity. Enzymes like MMP-9 cleave antibody hinge regions, unmasking cryptic C1q-binding sites and amplifying complement activation [68]. Anti-dengue antibody 2D22 exhibits 2.3-fold higher complement activation in infected tissues versus serum—a phenomenon linked to localized protease activity and cytokine levels within inflammatory microenvironments [69,70].

#### 2.2.2. The Central Role of Secretory Immunoglobulin A (sIgA) in Mucosal Immunity

sIgA constitutes the principal effector molecule of mucosal immunity, playing indispensable roles in preventing viral invasion. As respiratory and gastrointestinal tracts represent primary viral entry portals, sIgA establishes the primary defensive barrier through coordinated mechanisms—including multivalent antigen binding and transcytosis. This dynamic immunological barrier operates across multiple dimensions to suppress viral transmission and infection, while synergizing with local immune cells to enhance protection.

**Figure 4:**
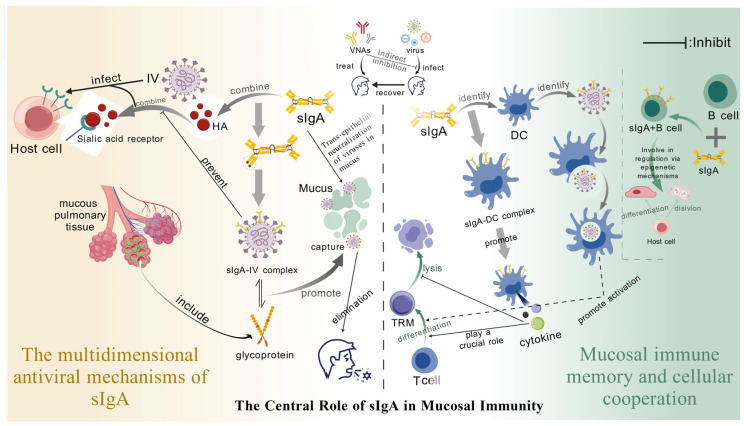
**The Central Role of sIgA in Mucosal Immunity.** (1) sIgA mucosal barrier: (i) the multivalent Fab cross-links the viral antigen, spatially blocking receptor binding; (ii) the immune complex is captured by mucus and expelled along with cilia oscillation; and (iii) pIgR mediates transepithelial transport, and intracellular neutralization of the released virus by infected cells. (2) sIgA-TRM memory: (i) binding to DC receptors induces IL-15, shaping the microenvironment for TRM differentiation; (ii) the immune complex-DC presentation drives the transformation of naive T cells into TRM; and (iii) epigenetic regulation maintains the long lifespan and continuous antibody secretion of mucosal sIgA + B cells.

Structurally, sIgA comprises two IgA monomers covalently linked by a joining (J) chain and noncovalently associated with secretory component (SC) derived from epithelial cells [71]. This unique tetrameric architecture provides multiple antigen-binding sites, enabling simultaneous engagement of viral epitopes to form extensive immune complexes. This multivalency both enhances binding avidity/specificity and establishes the structural foundation for sIgA’s multifaceted antiviral mechanisms. Critically, SC confers enzymatic stability by protecting sIgA from protease degradation in harsh mucosal environments, thereby extending its functional half-life [72]. Furthermore, SC may modulate sIgA interactions with viral particles and host cellular receptors, suggesting additional immunoregulatory functions beyond stabilization [73] (Figure 4).

##### The Multi-Dimensional Antiviral Mechanisms of sIgA

*Spatial blocking and mucus removal*

At mucosal surfaces, sIgA leverages its multivalent binding capability to tightly cross-link pathogens. This direct binding induces potent steric hindrance, effectively blocking the molecular recognition and interaction between pathogen surface ligands and host cell receptors [74]. For instance, sIgA can bind hemagglutinin on the influenza virus envelope, occluding its sialic acid receptor-binding sites. This steric shielding prevents viral attachment to respiratory epithelial cells, thereby blocking the critical initial step of infection [72]. This neutralization mechanism exhibits high specificity by targeting essential RBDs across diverse viruses. Crucially, it operates independently of complement activation or immune cell recruitment, enabling rapid deployment during the earliest stages of viral encounter to provide immediate, innate protection.

*Transepithelial neutralization*

Following synthesis by plasma cells in the lamina propria, sIgA binds the polymeric immunoglobulin receptor (pIgR) on the basolateral surface of epithelial cells. This binding initiates receptor-mediated endocytosis, facilitating transcytosis across the epithelial layer before apical secretion into mucosal surfaces [75]. Crucially, when transcytosing the infected epithelium, sIgA may bind intracellular viral particles, neutralizing them prior to virion release. This intracellular neutralization mechanism impedes cell-to-cell viral spread and restricts tissue dissemination [76], proving vital for controlling early-stage infection and preventing viremia.

Beyond classical transcytosis-mediated neutralization, emerging evidence indicates sIgA can enter infected cells through alternative pathways to exert intracellular antiviral effects [77,78]. Within endosomal compartments, sIgA binds viral nucleic acids or capsid proteins, inhibiting replication, transcription, and assembly [79]. This represents a paradigm shift: sIgA not only blocks extracellular viral entry but also targets intracellular pathogens, establishing a novel dimension of antiviral defense.

##### Mucosal Immune Memory Works in Synergy with Cells

The mucosal immune system can establish a complex and highly efficient defense network by generating sIgA and forming diverse immune cell populations. In this network, the formation of immune memory and the intercellular cooperation are crucial for maintaining the long-term immune homeostasis of the mucosa. The following will elaborate in detail on CD8^+^ tissue-resident memory T cells (TRM) and the epigenetic regulation of B cells.

*The synergistic effect of TRM and sIgA*

TRM cells, as a key component of mucosal immune memory, can remain in the tissue for a long time and quickly initiate the immune response upon encountering the pathogen again, providing immediate protection for the body. Recent studies have revealed that sIgA plays an indispensable indirect regulatory role in the differentiation of TRM cells [80]. On one hand, sIgA can bind to the specific receptors on the surface of mucosal DCs, through which this binding event activates the specific signaling pathways within DCs, prompting DCs to secrete a series of cytokines, such as interleukin-15 (IL-15) [80], which are crucial for the differentiation and maintenance of TRM cells [81]. On the other hand, the immune complexes formed by sIgA and pathogens can also be taken up by DCs, and after uptake, DCs process and present the pathogen-related antigens, thereby activating the differentiation of naive T cells into TRM cells [80].

A large number of experimental studies have shown that oral administration of engineered anti-rotavirus sIgA can significantly enhance the immune protection ability of the intestinal mucosa [82,83,84]. Rotavirus, as the main pathogen causing severe diarrhea in infants, seriously threatens the health of children [85]. Stephanie et al. demonstrated that after the mother ingested engineered sIgA, it could be effectively given to the infant through the breast milk route to inhibit the adsorption and infection of rotavirus on the infant’s intestinal mucosa, thereby reducing the severity and duration of diarrhea symptoms [84]. However, although this strategy shows promising application prospects, the specific protection time still needs further research. Future studies need to conduct long-term follow-up investigations and animal model experiments to clarify the duration of immune protection after oral administration of sIgA, and analyze the factors affecting the protection time, such as the dose of sIgA, the frequency of administration, the individual’s immune status, etc.

*Epigenetic regulation of B cells maintains mucosal immune stability*

During the mucosal immune response, the prolonged survival time of sIgA + B cells in the mucosal area is crucial for maintaining long-term immune memory and immune protection. Multiple studies have shown that this prolonged survival time is associated with various regulatory mechanisms, among which epigenetic regulation plays a key role [86]. Epigenetic regulation regulates gene expression without altering the DNA sequence, thereby influencing the function and fate of cells. For instance, Luo et al. found that the transcription factor ZBTB18 can promote the survival and functional maturation of memory B cells by inhibiting cell cycle genes, down-regulating pro-apoptotic factors, and blocking the germinal center retention factors; this study also pointed out that in sIgA + B cells, epigenetic mechanisms such as DNA methylation, histone modification, and non-coding RNAs are involved in regulating cell survival, proliferation, and differentiation [87].

For instance, the low DNA methylation status in the promoter region of specific genes may promote the expression of genes related to cell survival, thereby prolonging the survival time of sIgA + B cells in the mucosal area [88,89]. Additionally, the acetylation and methylation modifications of histones can also alter the chromatin structure and affect the transcriptional activity of genes, thereby regulating the function of B cells [89,90]. Although these studies have revealed the significant role of epigenetic regulation in mucosal immunity, the specific molecular mechanisms still require further exploration. Future research needs to utilize advanced molecular biology techniques, such as chromatin immunoprecipitation and genome-wide methylation sequencing, to identify the epigenetic modification sites and related regulatory factors involved in the survival regulation of sIgA + B cells, and to analyze their upstream and downstream signaling pathways, in order to provide a theoretical basis for a deeper understanding of the maintenance mechanism of mucosal immune memory.

## 3. The Mechanisms by Which Viruses Evade Neutralizing Antibodies

After analyzing the mechanism of action of VNAs, we further focused on the complex strategies of virus escape and antibodies. Increasing studies have shown that these intricate escape pathways not only weaken the efficacy of neutralizing antibodies but also pose severe challenges to the development of vaccines and antibody therapies [91,92]. Based on this background, this article reviews the escape mechanisms of viruses, including antigen variation, glycosylation shielding, conformational dynamics, epitope hiding, ADE, immune suppression and antibody interference, as well as escape mutations under the pressure of herd immunity, aiming to reveal the adaptability and evolutionary ability of viruses and provide directions for future antiviral research (Table 1).

### 3.1. Antigen Variation

#### 3.1.1. High-Frequency Point Mutations (Point Drift)

Viruses exploit the low-fidelity replication of RNA-dependent RNA polymerases (RdRps) or reverse transcriptases to accumulate point mutations under host immune pressure. These alterations in critical neutralizing epitopes compromise the antibody-binding capacity. For instance, the SARS-CoV-2 Omicron subvariant JN.1 harbors L455S and F456L mutations in its spike RBD, profoundly reducing neutralization titers against monoclonal antibodies S309 and REGN10987 [93]. Parallel evasion mechanisms operate in influenza A virus, where antigenic drift—driven by HA gene mutations—modifies viral antigenicity to escape antibody recognition [94,95]. Notably, monoclonal antibody studies targeting HA receptor-binding sites and flanking regions reveal both escape pathways [95] and unexpected epitope conservation across influenza subtypes [94].

#### 3.1.2. Antigenic Shift

Beyond antigenic shifts mediated by point mutations in influenza A virus’s HA/NA genes [95], viruses acquire novel epitopes through zoonotic transmission or reassortment, enabling complete evasion of established antibody responses. Nikolai et al. demonstrated that the H5N1-H1pdm reassortant incorporates the H1N1-derived Ca2 antigenic site into its HA protein, abrogating neutralization by antibodies targeting canonical H5N1 epitopes [96]. Concurrently, the SARS-CoV-2 XBC recombinant lineage—combining Omicron BA.2.75′s RBD mutations (e.g., K444T) with Delta’s NTD deletion (Δ143–145)—exhibits pronounced antigenic divergence from parental strains, evading monoclonal antibodies targeting ancestral RBD [97]. Separately, Dai et al. revealed that EV-D68 undergoes structural remodeling (e.g., VP1 T232K) to generate neotopes that resist cross-neutralization by antibodies against historical strains [98].

### 3.2. Glycosylation Shielding

Glycosylation shielding enables viruses to exploit host glycosylation machinery, adding glycan chains to key epitopes that create steric hindrance or charge masking to block antibody access. The HIV-1 Env “glycan shield” exemplifies this strategy: dynamic modifications at V3 loop sites N332 and N301 occlude the CDR H3 binding interface of broad-neutralizing antibody 10E8 [18,99]. Recent studies reveal that O-glycosylation clusters proximal to SARS-CoV-2 spike’s furin cleavage site—catalyzed by tandem GalNAc-transferases—modulate furin-mediated S protein cleavage, virus-like particle assembly, and viral infectivity in human lung cells [100]. This underscores how spike mutations alter host glycosylation regulation during SARS-CoV-2 evolution. Notably, Zhang et al. identified loss of N370 glycosylation as a driver of enhanced transmissibility in evolving SARS-CoV-2 lineages [101].

Historically, DENV epitomized glycosylation-mediated evasion. Fakhriedzwan et al. demonstrated that glycans at site N67 facilitate DENV attachment to DCs via DC-SIGN, promoting viral aggregation and infection [102]. Crucially, N67 glycosylation governs DENV infectivity and morphogenesis. Juan et al. found it enhances infection efficiency in immature DCs and mediates E protein secretion during virion maturation [103]. In mammalian cells, N67 ablation severely impairs E protein secretion—an effect less pronounced in mosquito cells—highlighting host-specific functional divergence linked to viral adaptation. Moreover, N67 deletion selectively reduces infectivity in mammalian systems without compromising mosquito-cell entry, implicating this site in zoonotic transmission.

### 3.3. Dynamic Configuration

Certain viral surface proteins exploit conformational rearrangements or dynamic fluctuations to occlude neutralizing epitopes, exposing them only during specific infection stages—a phenomenon termed conformational masking. In RSV, antigenic site Ø (residues 62–69) of the F protein undergoes β-sheet restructuring during pre to postfusion transition, preventing stable binding by antibody nirsevimab [104]. Similarly, Ebola virus GP’s RBD adopts a closed conformation in its unliganded state, exposing conserved epitopes solely upon NPC1 receptor engagement to evade closed-state neutralizing antibodies [105]. Recently, the SARS-CoV-2 spike exemplifies this paradigm: its NTD exhibits “dynamic breathing” when unbound to ACE2 [106], while recurrent RBD/NTD mutations (R346T, K417N/T, L452R, N460K, E484A/K/Q, N501Y) in Omicron subvariants collectively drive substantial antibody evasion and immune escape [107].

### 3.4. Physical Masking and Molecular Camouflage

Beyond molecular-level adaptations, viruses employ physical masking or molecular camouflage strategies to occlude critical epitopes. Hepatitis C virus (HCV) utilizes host-derived apolipoprotein E to form complexes that sterically shield the E2 protein’s CD81-binding domain, impeding neutralization by antibodies like AR3A [108]. Epstein–Barr virus (EBV) deploys soluble monomeric forms of gp350 that competitively bind neutralizing antibody 72A1, reducing antibody targeting of viral particles [109]. Notably, the Zika virus NS1 protein, adsorbed onto host cell membranes, exploits phosphatidylserine-mediated “eat-me” signals to camouflage virions, enabling evasion of antibody ZIKV-117 recognition [110]; Certain viruses can secrete non-infectious viral particles or proteins as molecular “decoys”, which deplete VNAs before they encounter intact virions [111]. For instance, HBV secretes subviral particles—HBsAg—that efficiently bind and neutralize specific antibodies, thereby substantially reducing the concentration of functional antibodies and consequently diminishing their neutralizing capacity [112].

### 3.5. ADE

As mentioned previously, sub-neutralizing or non-neutralizing antibodies can paradoxically enhance viral internalization via FcγR-mediated mechanisms—a phenomenon termed ADE. In secondary DENV infections, antibodies at sub-neutralizing concentrations (e.g., 2D22) augment viral replication within monocytes through FcγRIIa-dependent endocytosis [47]. For SARS-CoV-2 Omicron BA.2.86, the spike F486P mutation induces Fc conformational changes that increase affinity for FcγRIIIa, elevating viral loads in alveolar macrophages [113,114]. Studies reveal that HIV-1 antigens on infected cells engage host FcγRs, not only promoting viral entry but also dysregulating phagocytic and antigen-presenting functions in macrophages and DCs—thereby suppressing immune responses [115]. Furthermore, ADE facilitates cell-to-cell spread of HIV-1: following FcγR-mediated endocytosis, virions replicate within immune cells and disseminate while circumventing immune surveillance. These mechanisms collectively facilitate viral persistence by evading immune recognition and clearance [115].

### 3.6. Immunosuppression and Antibody Interference

Viruses subvert antibody-mediated immunity through immune suppression and antibody interference mechanisms, often by hijacking host immunoregulatory pathways or secreting inhibitory proteins. Human cytomegalovirus encodes Fc receptors TRL11/TRL12 that sequester IgG Fc regions, both blocking FcγR engagement and promoting antibody degradation via endocytosis-mediated interference with FcγRn recycling [116]. Similarly, HSV-1 deploys the gE/gI complex—a structural mimic of host Fc receptors—the extracellular gE domain of which binds antibody Fc regions to mediate endocytosis and retrograde trafficking to lysosomal compartments [117,118]. Additionally, the SARS-CoV-2 ORF8 protein suppresses major histocompatibility complex class I (MHC-I) expression, reducing viral antigen presentation and indirectly impairing neutralizing antibody effector functions [119].

### 3.7. Escape Mutations Under Herd Immunity Pressure

Under herd immune pressure such as vaccine or natural infection, some viruses can accumulate escape mutations through positive selection. Recent studies have shown that the HA receptor-binding mode of human H3N2 has evolved further [120]. The results of X-ray crystallography analysis showed that two natural mutations, G186D and D190N, coevolved at the HA receptor-binding site and coordinated to drive the evolution of the HA receptor-binding mode. This coevolution is due to an epigenetic interaction between G186D and D190N, which together can maintain high-affinity binding to α2, 6-linked sialoglycosides, whereas alone they impair this binding. In addition, the evolution of this receptor-binding mode is accompanied by changes in the antigenicity of HA and affects the compatibility of the egg-adaptive mutation L194P, which is common in vaccine production [120].

Interestingly, the evolutionary trajectory of SARS-CoV-2 once again validates the classic “mutation-escape” strategy. In the case of the JN.1 variant, the P681R substitution near the furin cleavage site not only improves the pre-activation efficiency of the spike protein, but also successfully elicits neutralizing antibodies targeting the original site [114,121]. E484K almost became a common tag of several variants of concern (B.1.1.7, R.1, B.1.351, etc.), and the charge turnover of this site significantly weakened the recognition ability of VNAs to RBD, resulting in a general decrease in neutralization activity [122]. Moreover, the application of therapeutic monoclonal antibodies exerts significant selective pressure on viruses. During extended mAb therapy in immunocompromised patients, sustained viral replication facilitates the emergence of escape variants. This phenomenon has been extensively documented in SARS-CoV-2 antibody treatments, exemplified by Omicron sub-lineages such as BQ.1.1, the mutations of which enable the virus to evade the effects of multiple neutralizing antibodies [123,124,125].

The synergistic upgrading of transmission and immune escape is also reflected in mutations such as N501Y, L452R, and T478K: N501Y enhances the binding of ACE2 and expands the cross-species infection spectrum [122]. L452R/T478K appeared repeatedly in Delta and Omicron, and the multiple substitutions of Omicron RBD made its affinity three to five times higher than that of the original strain, accompanied by a surge in breakthrough infection [126,127]. Experimental data showed that the neutralization titer against Delta has dropped to 1/5.8 of the original level, while the neutralization titer against BA.1 and BA.2 plunged 17.4 times and 14.2 times, respectively, suggesting that the antibody barrier induced by the current vaccine is continuously eroded [127].

### 3.8. The Synergy of Escape Strategies

Beyond individual escape mechanisms, viruses synergistically deploy combinatorial strategies to achieve high-efficiency immune evasion during evolution. SARS-CoV-2 variants exemplify this through concurrent mutations: F486P in the RBD enhances ACE2 affinity, Δ144–145 deletion in the NTD masks immunodominant epitopes, and D1139H in the S2 subunit retards fusion kinetics—collectively enabling escape from monoclonal antibody cocktails [128,129]. Furthermore, key N-glycosylation sites on the SARS-CoV-2 spike protein stabilize its receptor-accessible “up” conformation to promote ACE2 engagement while simultaneously impeding antibody neutralization. Crucially, the steric bulk of glycans introduces a pause during conformational transitions, allowing extended exposure of the fusion peptide for host membrane capture and enhanced infectivity [130]. Conversely, mutations such as N165A and N234A shift the conformational equilibrium toward the “down” state, reducing fusogenicity and increasing vulnerability to immune recognition [130].

Collectively, viruses evade VNAs through multifaceted and mechanistically complex strategies that amplify viral diversity while posing challenges to existing vaccines and antibody therapies. Future research must elucidate the molecular foundations of these escape pathways to develop pan-variant neutralizing antibodies and universal vaccines resilient to viral evolution.

## 4. Perspectives and Closing Remarks

Current advances in VNAs development have ushered in an era of profound convergence among computational biology, immune engineering, and clinical medicine. This technological synergy not only accelerates the generation of high-potency antibodies but also extends the frontiers of precision medicine through multidisciplinary integration, providing transformative tools to counter viral evolution while addressing personalized therapeutic imperatives.

### 4.1. Rational Design of Antibodies Driven by Computational Biology

AI-driven computational tools leveraging high-fidelity modeling are redefining antibody design paradigms. Deep generative models such as IgLM simulate the sequence landscape of natural antibody repertoires to predict CDR conformational diversity, enabling the successful engineering of broad-spectrum neutralizing antibodies including SA55—which targets SARS-CoV-2 Omicron subvariants (e.g., XBB.1.5) through synergistic engagement of RBD and NTD epitopes on the spike protein, substantially curtailing viral escape [131]. Furthermore, AlphaFold-Multimer achieves atomic-level precision in predicting antibody-antigen complexes, exemplified by its resolution of dynamic binding interfaces between HIV Env trimers and broadly neutralizing antibody PGT145, thereby guiding structure-informed CDR loop optimization [132,133]. Meanwhile, molecular dynamics simulations elucidate functional consequences of antibody structural plasticity: strategic enhancement of rotational freedom in the Fab domains of Ebola virus antibody ADI-15878 boosted neutralization potency 3-fold [134]. Notably, the deep learning framework EpitopeVec identifies cryptic conserved epitopes on coronavirus spike proteins through analysis of million-scale viral epitope datasets, unveiling novel targets for universal antibody development [135].

### 4.2. Immunoengineering Empowers the Innovation of Antibody Function and Delivery

Breakthroughs in synthetic biology and protein engineering are expanding the functional boundaries of antibodies. Multi-specific antibody designs have significantly improved the antiviral spectrum. For example, the two-epitope antibody CoV-X2 targets a conserved region of the S2 subunit of the SARS-CoV-2 spike protein and can neutralize a variety of variants, including JN.1 [136]. The glycosylation reprogramming technique [137] greatly enhances the affinity of IgG1 with FcγRIIIa by knocking out the Asn297 core fucose [138].

In terms of delivery system, the combination of nanoparticles and mucosal delivery technology achieves local immune protection: liposomal nanoparticles loaded with sIgA can block the binding of the influenza virus HA protein to host receptors in the respiratory mucosa and activate memory T cell responses by nasal administration [139,140]. However, the mRNA-LNP platform can encode bispecific antibodies (such as Flu-RSV BiAb), which can achieve double repeat coverage of mucosal and systemic immunity through transient expression in vivo [141].

### 4.3. Individualized Treatment Strategies in the Context of Precision Medicine

Breakthroughs in single-cell sequencing and high-throughput screening technologies have propelled antibody development from population-level approaches to single-cell precision. Single-cell sequencing enables the simultaneous resolution of full-length antibody sequences from thousands of immune cells in a single experiment, directly capturing natively paired heavy- and light-chain genes. This advancement compresses the discovery timeline for candidate antibodies from months to weeks [142,143]. In the case of Ebola, researchers efficiently isolated mAb114-M2 from convalescent peripheral blood; this antibody targets a conserved hydrophobic pocket at the base of the viral GP protein, neutralizing 99% of known strains—demonstrating the dual advantages of efficiency and breadth inherent to single-cell strategies [144]. Similarly, HCV non-structural proteins (NS3/4A/4B/5B) were deconstructed into multiplexed antigen ‘modules’ capable of activating CD8^+^ T cells. When delivered via viral vectors or DNA vaccines, these modules elicited significantly superior T cell responses compared to single antigens, showcasing synergistic vaccine design at the ‘single-genome’ level [145]. Furthermore, synthetic biology has revolutionized antibody production precision. Yeast genome-reprogramming platforms optimize glycosylation pathways, reducing production costs for complex glycoform antibodies by an order of magnitude and dramatically enhancing accessibility to personalized neutralizing antibodies [146,147].

Collectively, these cases delineate an end-to-end precision R&D pipeline spanning discovery, design, and production. Future antibody development may thus adopt a tripartite strategy: first, leveraging single-cell sequencing and high-throughput screening to identify optimal candidates; second, broadening immune coverage through multipathogen antigen combinations; and finally, utilizing synthetic biology tools like yeast glycoengineering for cost-effective manufacturing. This trajectory will usher neutralizing antibodies into a new era of individualized therapy driven by ‘single-monoclonal, single-cell, and single-genome’ paradigms.

### 4.4. Future Directions and R&D Challenges

In summary, the field of VNAs is advancing rapidly toward achieving greater breadth, extended durability, and precision targeting. However, each technological innovation necessitates rigorous optimization and validation. Critical forward-looking questions remain unresolved: How can we shift from reactive antibody development to proactive design that anticipates viral evolutionary trajectories? For specific pathogens (e.g., HIV and influenza viruses), how do we strike an optimal balance between Fab-mediated neutralization potency and Fc-driven effector functions? Beyond efficacy, how can emerging production platforms (e.g., mRNA, yeast expression systems) overcome cost and manufacturing barriers to ensure global accessibility? Substantial challenges persist across three domains:

(i) Biological challenges: Rapid viral mutations and antigenic diversity demand iterative antibody design. Before the arrival of new variant viruses, can we shift from reactive antibody design (responding to new variants) to proactive/predictive design anticipating viral evolution (AI-guided approaches)? With the crossdisciplinary integration and the development of AI technology, VNAs are gradually moving from “passively following virus evolution” to a new paradigm of “active prediction-active design”. Generative AI platforms (e.g., EVEscape, AlphaFold-Multimer, RFdiffusion) now enable proactive prediction of epitope evolution and antibody sequences months before new variants emerge, facilitating ‘zero-lag’ antibody library generation [148]. Integrated with microfluidics-coupled B-cell single-cell omics and CRISPR editing, these AI designs undergo functional validation within 2–3 weeks, accelerating the design–testing cycle [149].
(ii) Technological and translational challenges: The development of antibody drugs has long faced challenges such as large-scale production, long-term safety, and cost control [150]. During the process of implementation, significant progress has been made in existing technologies. For instance, the half-life engineering (e.g., YTE mutation, as in nirsevimab) has extended the dosing interval from weeks to months, significantly reducing the medical burden in scenarios like virus prevention for premature infants [151], while new delivery systems such as combined dosing regimens, micro-needle arrays, and nasal dry powder inhalation devices are gradually replacing traditional intravenous infusion, enhancing the accessibility of drugs during the pandemic. Moreover, emerging production platforms also demonstrate great potential. For instance, research has shown that the mRNA in vitro transcription-liposome encapsulation method can achieve “digital” antibody production within a short period of time [152]. The economic advantages of the monoclonal antibody production platform are prominent, and in the future, it will provide more affordable localized production solutions for low-income countries.
(iii) Logistical and regulatory challenges: Regulatory frameworks must evolve to address novel modalities. mRNA-encoded antibodies and highly engineered formats (e.g., multi-specifics, Fc-modified antibodies) pose unique evaluation complexities for agencies like the FDA and EMA. Overcoming these demands integrating computational biology, synthetic immunology, and clinical insights.

The future of VNAs hinges on systemic integration. Synergizing AI-driven epitope prediction, programmable manufacturing, adaptive regulatory policies, and global health equity strategies will forge an efficient ‘design-produce-deliver-reimburse’ continuum. This promises to transform VNAs from bench innovations into accessible public health resources.

## Figures and Tables

**Table 1:** The mechanisms by which viruses evade neutralizing antibodies.

Escape Mechanisms	Specific Modalities	Action Principle	Influence
Antigen variation	High-frequency point mutation (antigenic drift)	The virus accumulates point mutations through low-fidelity replication, changing the amino acid sequence of key neutralizing epitopes and weakening antibody binding ability.	Weakening the effectiveness of neutralizing antibodies increases the difficulty of developing vaccines and antibody therapies.
	Antigenic shift	Viruses acquire completely new epitopes through genome recombination or genotype transformation, thereby evading existing antibody responses.	It deactivates existing antibodies and increases the risk of transmission and infection.
Glycosylation shielding	Glycosylation	Viruses add glycan chains around key epitopes to form steric or charge shielding that prevents antibody binding.	Prevent antibody binding and enhance the ability of the virus to escape.
Dynamic configuration	Tablet hiding and dynamic exposure	The viral surface protein hides neutralizing epitopes through conformational rearrangement or dynamic fluctuations and is only exposed at specific stages of infection.	It is difficult to be continuously recognized by antibodies, increasing the chance of immune escape.
Physical masking and molecular camouflage	Physical shielding epitopes and the evasion of antibody recognition	Viruses use external molecules to physically mask the epitopes, or they use molecular signals to deceive, thereby resisting antibody recognition.	Block antibody recognition and reduce neutralization efficiency.
ADE effect	Antibody-dependent enhancement	When inefficient antibodies are present, the virus particles can passively enter the cells through the FcγR pathway to enhance infection.	Enhance the ability to infect and make antibody therapy ineffective.
Immunosuppression and antibody interference	Competition of immunoreactive agents or antibodies	Viruses reduce antibody effectiveness by secreting immunosuppressants or inducing antibody competition.	Interferes with immune response and weakens antibody efficacy.
Escape mutations under herd immunity pressure	Mutations under population immunization selection pressure	Viruses produce adaptive mutations under the pressure of herd immunity to escape herd immunity response.	Increases the risk of transmission and infection, challenging herd immunity strategies.

## Data Availability

No new data were created or analyzed in this study. Data sharing is not applicable to this article.

## Abbreviations

The following abbreviations are used in this manuscript:

**Abbreviations**	**Full names**
VNAs	Antiviral neutralizing antibodies
ADE	Antibody-dependent enhancement
cryo-EM	Cryo-electron microscopy
CDR	Complementarity-determining region
RBD	Receptor-binding domain
ACE2	Angiotensin-converting enzyme 2
IV	Influenza virus
HA	Hemagglutinin
WNV	West Nile virus
NTD	N-terminal domain
BsAb	Bispecific antibody
RSV	Respiratory syncytial virus
HEV	Hepatitis E Virus
RhV	Rhinovirus
SAXS	Small-angle X-ray scattering
cryo-ET	Cryo-electron tomography
6-HB	Six-helix bundle
ADCC	Antibody-dependent cell-mediated cytotoxicity
CD16	FcγRIII
ADCP	Antibody-dependent cellular phagocytosis
MAC	Membrane attack complex
CDC	Complement dependent cytotoxicity
sIgA	Secretory immunoglobulin A
DC	Dendritic cell
TRM	Tissue-resident memory T
non-NAbs	Non neutralizing antibodies
weak-NAbs	Weak neutralizing antibodies
NK	Natural killer
FcμR	Fcμ receptor
SC	Secretory component
pIgR	Polymeric immunoglobulin receptor
TRM	Tissue-resident memory T cells
IL-15	Interleukin-15
RdRps	RNA polymerases
HCV	Hepatitis C virus
EBV	Epstein–Barr virus
MHC-I	Major histocompatibility complex class I
